# A Novel Approach to Ovarian Cancer Diagnosis via CT Imaging: GPT-4o-Driven Automated Feature Recognition and Validation in Clinical Settings

**DOI:** 10.1245/s10434-026-19248-2

**Published:** 2026-02-17

**Authors:** Shimin Zhang, Qiuyang Hou, Mufei Ding, Yuming Zhu, Gang Dai, Zhao Lu, Zhuonan Liu, Bosinan Chen, Xiaogeng Li, Jingyi Liu, Kexue Deng, Jiangdian Song, Xin Zhou

**Affiliations:** 1 Department of Obstetrics and Gynecology, Shengjing Hospital of China Medical University, Shenyang, Liaoning China; 2 Department of Radiology, The First Affiliated Hospital of University of Science and Technology of China (USTC); Division of Life Sciences and Medicine, USTC, Hefei, Anhui China; 3 School of Health Management, China Medical University, Shenyang, Liaoning China; 4 Department of Radiology, Shengjing Hospital of China Medical University, Shenyang, Liaoning China; 5 The First Hospital of China Medical University, Shenyang, Liaoning China; 6 School of Intelligent Medicine, China Medical University, Shenyang, Liaoning China

**Keywords:** Ovarian cancer, Artificial intelligence, Large language models, Diagnosis, Medical imaging

## Abstract

**Background:**

Accurate non-invasive diagnosis of early-stage ovarian cancer remains challenging because of the limited number of biomarkers. Although artificial intelligence algorithms show promise for ovarian cancer diagnosis, their reliance on specialized engineering knowledge hinders their accessibility. The recent emergence of visual large language models such as GPT-4o has further expanded the potential of AI in this domain.

**Methods:**

GPT-4o was trained to automatically recognize ovarian lesions, report key computed tomography (CT) features of ovarian lesions, and make a benign or malignant diagnosis based on these features. Radiologists and gynecologic oncologists independently reviewed the GPT-4o reports and evaluated GPT-4o's performance.

**Results:**

GPT-4o achieved diagnostic accuracies of 80.80%, 79.14%, and 93.33% in the three datasets. Its performance surpassed that of gynecologic oncologist with 10 years of experience but was inferior to that of gynecologic oncologist with 16 years of experience and radiologists with ≥ 7 years of experience. The clinician-rated reliability in detecting the four key CT features was 4.22/5.00 for cyst wall and septum status; 4.24/5.00 for nodular or papillary protrusions; 4.30/5.00 for density and enhancement distribution; and 4.25/5.00 for cystic-solid characteristics. The use of GPT-4o increased the accuracy of radiologist and gynecologic oncologist diagnoses by 1.96% and 10.50%, respectively.

**Conclusions:**

GPT-4o identifies the key CT features of ovarian cancer and achieves promising diagnostic accuracy with high-quality diagnostic evidence.

**Supplementary Information:**

The online version contains supplementary material available at 10.1245/s10434-026-19248-2.

Ovarian cancer is the eighth most common cancer in women and is a significant global health concern, accounting for 3.4% of new cancer cases and 4.8% of cancer-related deaths in 2022.^1^ Ovarian cancer has the highest mortality rate among all gynecological cancers because patients often present at an advanced stage, as early symptoms are non-specific and reliable early detection biomarkers are lacking.^2,3^ Consequently, 55% of ovarian cancers are initially diagnosed as metastatic, with a 5-year survival rate of 31.4%.^4^ Early detection significantly improves survival rates, specifically when the tumor is confined to the ovaries or fallopian tubes (International Federation of Obstetrics and Gynecology Stage I), with disease having a 5-year survival rate of > 90%.^5^ Therefore, the development of accurate early diagnostic approaches is crucial for improving prognosis.

For patients with suspected ovarian cancer, surgical resection followed by histopathological examination is regarded as the diagnostic gold standard.^6^ However, definitive pathological confirmation can only be obtained after surgery, highlighting the critical need for accurate preoperative assessment to guide clinical decision-making. In this context, radiological imaging, particularly abdominal/pelvic computed tomography (CT), has emerged as a crucial tool for the initial assessment of patients with risky pelvic masses or related symptoms.^7^ Although ultrasound and magnetic resonance imaging (MRI) play important and complementary roles in the evaluation of adnexal masses and may provide more detailed tissue characterization in certain clinical scenarios, abdominal and pelvic CT is routinely performed in patients with suspected ovarian cancer, particularly for disease staging and assessment of extra-ovarian involvement. Accurate interpretation of specific imaging features on pelvic CT, including cyst wall and septum status, presence and characteristics of nodular or papillary protrusions, density and enhancement distribution, and cystic-solid composition (cystic, solid, or mixed cystic-solid), is essential for the early detection of ovarian cancer.^8–11^ These imaging features represent radiological descriptors commonly used in clinical practice to differentiate benign from malignant ovarian masses. However, this approach relies heavily on radiologist experience and is susceptible to interobserver variability, potentially introducing uncertainties in clinical decision-making.^12^ Recent advancements in artificial intelligence (AI), particularly in the field of computer vision, offer promising avenues for overcoming these limitations.^13^ By characterizing the semantic features within CT images, AI algorithms can assist clinicians in discerning subtle differences in imaging characteristics, thereby aiding the early diagnosis of ovarian cancer.^14,15^ The recent emergence of visual large language models such as GPT-4o has further expanded the potential of AI in this domain.^16,17^ These models leverage their capacity for video understanding to mimic radiologists in identifying abnormalities in medical images, achieving a level of human-like diagnostic capability without needing specialized neural networks and relying on high-performance computing hardware, which are clinically scarce resources.^18–20^ This advancement has the potential to translate the AI-assisted diagnosis of ovarian cancer into clinical practice.^21^ However, the ability of GPT-4o to specifically identify CT signs of ovarian cancer and improve the accuracy of clinician diagnosis remains to be explored.

Therefore, this study aimed to evaluate the potential of GPT-4o for the automated diagnosis of early-stage ovarian cancer based on CT images. In this study, we assessed the model's ability to automatically identify the high-risk CT features in ovarian masses associated with malignancy and determined the ability of GPT-4o in assisting the diagnosis of early-stage ovarian cancer for both radiologists and gynecologic oncologists. This feature-based evaluation strategy enables an interpretable comparison of GPT-4o–generated findings with those identified by expert radiologists and gynecologic oncologists.

## Methods

### Study Design

**Fig. 1:**
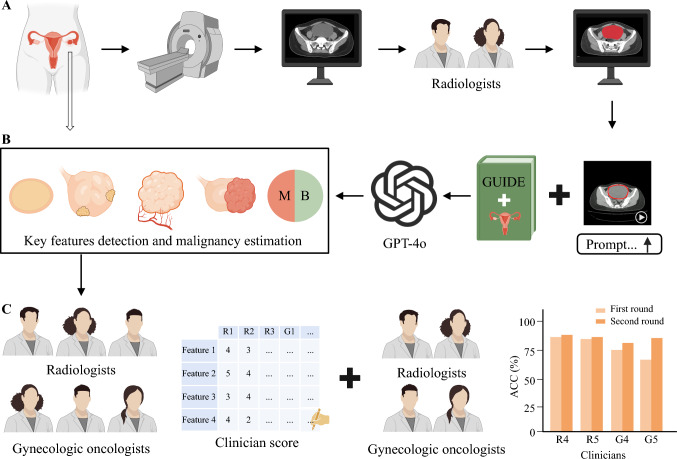
Study flowchart. **A** Patients’ pelvic computed tomography (CT) images were acquired from the picture archiving and communication system and converted to video. **B** The CT video and the clinical guidelines were then fed to GPT-4o for identification of ovarian mass image features and prediction of the probability of ovarian mass malignancy. **C** The results were evaluated by three radiologists and three gynecologic oncologists. Further, a test–retest experiment was conducted to evaluate the diagnostic improvement with GPT-4o assistance. Some icons in the figure were created with BioRender.com

Patients with pathologically confirmed early-stage ovarian cancer and those with noncancerous ovarian pathologies were included. Data were collected from two institutions between July 2018 and June 2024. Additionally, data from The Cancer Imaging Archive (TCIA) dataset were included.^22^ A three-step approach was used. First, videos were created from pre-treatment thin-section CT images of each patient. These videos, along with prompts, were inputted into GPT-4o to identify the key features of ovarian lesions and generate a diagnosis (benign or malignant) based on these features. A textual report outlining the identified features was required as the GPT-4o diagnostic evidence. Second, the diagnostic accuracy of GPT-4o was determined by comparing it with that of pathological testing. The reliability of the identified features was assessed by six radiology and gynecologic oncology specialists. Finally, to evaluate the improvement in diagnostic accuracy with GPT-4o assistance, four additional specialists (two radiologists and two gynecologic oncologists) independently reviewed the CT images of all patients in two rounds with and without GPT-4o output, separated by a 2-week washout period. The workflow is presented in Fig. 1.

### Patients

Patients from the two participating hospitals were enrolled based on the following inclusion criteria: (1) age ≥ 18 years and complete demographic data, (2) availability of pre-treatment thin-section pelvic CT images (with or without contrast enhancement), (3) presence of a distinct pelvic adnexal mass on CT imaging (minimum diameter 10 mm), and (4) definitive pathological diagnosis of either stage I ovarian cancer or a benign condition. The exclusion criteria were as follows: (1) insufficient image quality, (2) previously diagnosed cancer, (3) systemic inflammation and other major diseases, (4) other masses of non-ovarian origin, and (5) both benign and malignant ovarian lesions in the same patient.

For patients in TCIA dataset, those with pathology results from The Cancer Genome Atlas Ovarian Cancer (TCGA-OV) dataset were identified. From this group, patients with pathologically confirmed stage I and stage II disease with corresponding CT images were selected (because of limited data samples), forming the external test dataset of patients with early-stage ovarian cancer.

### CT Image Pre-processing

The pre-treatment CT images were reviewed by two experienced specialists (a gynecologic oncologist and a radiologist) with at least 10 years of experience. After reaching a consensus on the primary lesion boundary, the lesion was manually segmented using the ITK-SNAP software (version 3.6.0; http://www.itksnap.org), which has been previously applied for CT-based segmentation of ovarian masses in radiomics studies.^23,24^ In patients with multiple primary lesions, each lesion was individually segmented. Subsequently, the pelvic CT images were compiled into sequential videos using Python, adhering to a caudo-cranial (bottom-up) sequence. The window width and level were fixed at 400 and 35, respectively, for all the scans. The resulting CT videos had a resolution of *512×512×N* pixels, with the number of frames (*N*) corresponding to the number of CT slices. We compared several frame-rate settings (5, 10, 15, and 20 frames per second) on a pilot validation subset and found that 5 frames per second yielded the highest lesion-recognition accuracy with GPT-4o; this rate was therefore adopted for all videos. All personal identifying information from the CT videos was removed before input into GPT-4o to ensure privacy protection.

Segmentation masks delineated by two specialists were applied to the corresponding slices in the CT images. Preliminary testing revealed that GPT-4o could accurately identify lesion boundaries when the boundaries were marked in red with a width of five pixels on the slices (Supplementary Fig. S1). The CT videos and associated information required to reproduce this study are available in Mendeley Data: 10.17632/r3bxp8mskj.1

### GPT-4o Evaluation of Ovarian Lesions

The established clinical criteria for diagnosing ovarian cancer based on CT imaging features according to the current clinical guidelines and related studies^8–11^ were provided to GPT-4o (Supplementary A1). Subsequently, CT videos (with red marking of lesion boundaries), along with the corresponding radiological reports and pathological results, of 10 patients (five patients each with pathologically confirmed stage I ovarian cancer and benign tumors, respectively) randomly selected from the first participating hospital were inputted into GPT-4o for 10-shot training.^25^ This training process familiarized GPT-4o with the differences in CT imaging features between benign and malignant ovarian lesions and generated textual prompts for subsequent experiments.

The detailed prompts used for GPT-4o diagnosis are provided in Supplementary A2. The age and lesion size for each patient was provided to GPT-4o. The model was then instructed to perform the following tasks: (1) identify the ovarian mass region marked in red in the CT video across consecutive slices and (2) analyze the identified ovarian mass for the following clinically confirmed key CT features: cyst wall and septum status, presence and characteristics of nodular or papillary protrusions, density and enhancement distribution, and cystic-solid composition (cystic, solid, or mixed cystic-solid). These four CT features are key indicators for differentiating between benign and malignant ovarian masses based on the clinical guidelines and previous studies.^8–11^ Based on the identified features, GPT-4o was then used to estimate the probability of a lesion being malignant or benign. Finally, GPT-4o provided its output, including the identified lesion area for visualization, detected image features, and diagnoses.

### Clinician Evaluation of GPT-4o's Report

Six clinicians (three radiologists and three gynecologic oncologists) were recruited to diagnose ovarian lesions based on CT images and to evaluate the reliability of GPT-4o reports. R1, R2, and R3 (radiologists) had 22, 8, and 7 years of experience, respectively, and G1, G2, and G3 (gynecologic oncologists) had 16, 10, and 3 years of experience, respectively. Each clinician independently reviewed the CT images and basic patient information (the same information provided to GPT-4o). They were then asked to describe the four CT features mentioned above and record their diagnoses. Subsequently, they were provided with the output of GPT-4o and required to rate the concordance between their feature descriptions and those of GPT-4o using a 5-point Likert scale (1: completely incorrect, 2: more incorrect than correct, 3: equally correct and incorrect, 4: more correct than incorrect, and 5: completely correct).

To further assess the clinical utility of GPT-4o output, all six clinicians evaluated the information provided by the model. This evaluation focused on three domains^26^: (1) the extent of inappropriate content (1 point for “present, substantial clinical significance,” 2 points for “present, little clinical significance,” and 3 points for “none”), (2) extent of missing content (scored similarly to inappropriate content), and (3) likelihood of possible harm (1 point for “high,” 2 points for “moderate,” and 3 points for “low”).

### Test–Retest Experiment

The test–retest experiment consisted of two parts. First, to determine the reproducibility of GPT-4o diagnosis, 50 patients were randomly selected from the included datasets. At 2 weeks after the initial analysis, the same CT videos and prompts were inputted into GPT-4o based on the same condition. The diagnostic results and specific feature descriptions from both analyses were recorded. The reproducibility of the diagnosis (binary classification: malignant or benign) was directly compared between the two outputs. The reproducibility of the CT feature descriptions was subjectively assessed by two gynecologic oncologists, who assigned a score from 0 to 100% to each of the four features. The average score of the two specialists was used as the final reproducibility score for the GPT-4o feature descriptions.

Second, to evaluate the potential of GPT-4o to improve diagnostic accuracy, another test–retest experiment was conducted. This step involved two additional radiologists (R4 and R5) with 9 and 11 years of experience and two additional gynecologic oncologists (G4 and G5) with 6 and 2 years of experience, respectively. These clinicians first diagnosed patients based on CT images and baseline information (without GPT-4o assistance). After a 2-week washout period, they reviewed the data again, with the diagnostic results and feature descriptions provided by GPT-4o. By comparing the diagnostic accuracy between the two rounds, this study determined whether GPT-4o assistance could improve the accuracy of ovarian cancer diagnoses by both radiologists and gynecologic oncologists.

### Statistical Analysis

Area under the receiver operating characteristic curves (AUCs) were used to evaluate the accuracy of GPT-4o diagnoses compared with that of pathological results. Differences in AUCs between GPT-4o and clinician diagnoses were computed using the DeLong test.^27^ Diagnostic performance was assessed based on accuracy, specificity, and sensitivity, and all statistical calculations were performed with 95% confidence intervals (CIs). The agreement in feature detection between GPT-4o and the six clinicians was assessed using Likert scale scores, with median scores and interquartile ranges (IQRs), as well as mean scores and standard deviations (SDs). Weighted kappa (κ) was used to evaluate inter-clinician agreement on the scores, and the values were interpreted as follows: 0.21–0.40, fair; 0.41–0.60, moderate; 0.61–0.80, substantial; 0.81–1.00, excellent.^28^

All experiments were conducted using GPT-4o, accessed through the chat interface of the official OpenAI website, which allowed file uploads. All statistical analyses were performed using R software (version 4.0.3; R Foundation for Statistical Computing). A two-sided *p*-value of < 0.05 was considered significant.

## Results

### Patient Characteristics

**Fig. 2:**
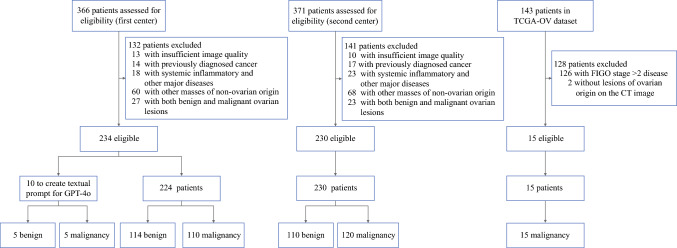
Patient enrollment flowchart. CT, computed tomography; FIGO, International Federation of Gynecology and Obstetrics; TCGA-OV, The Cancer Genome Atlas Ovarian Cancer

Overall, 479 patients were included (Fig. 2). In addition to the 10 patients for GPT-4o memory training, 224 patients (mean ± SD age 49.95 ± 13.75 years) from the first center, 230 patients (mean age 48.42 ± 15.46 years) from the second center, and 15 patients (mean age 66.53 ± 12.05 years) in the TCGA-OV dataset were included. The patient characteristics are shown in Table 1. Overall, 82 (17.12%) patients had multiple primary lesions, and 397 (82.88%) patients had a single primary lesion. The mean ± SD lesion size was 9.23 ± 5.32 cm.

**Table 1:** Patient characteristics

Characteristic	First center	Second center	TCGA-OV
Patients	224	230	15
Age, years	49.95 ± 13.75	48.42 ± 15.46	66.53 ± 12.05
Range	21–79	18–89	48–81
Menopausal status			
Premenopausal	90 (40.18)	109 (47.39)	NA
Postmenopausal	134 (59.82)	121 (52.61)	NA
Lesion diameter, cm	8.00 ± 4.69	10.30 ± 5.68	11.16 ± 4.37
Range	1.63–27.07	2.61–35.89	6.13–20.82
Histological subtype			
Benign	114	110	NA
Epithelial	31 (27.19)	64 (58.18)	NA
Serous	12 (10.53)	22 (20.00)	NA
Mucinous	13 (11.40)	21 (19.09)	NA
Other	6 (5.26)	21 (19.09)	NA
Non-epithelial	83 (72.81)	46 (41.82)	NA
Sex cord-stromal	56 (49.12)	10 (9.09)	NA
Germ cell	25 (21.93)	28 (25.45)	NA
Other	2 (1.75)	8 (7.27)	NA
Malignant	110	120	15
Epithelial	67 (60.91)	100 (83.33)	15 (100.00)
Serous	23 (20.91)	70 (58.33)	15 (100.00)
Mucinous	5 (4.55)	6 (5.00)	NA
Endometrioid	17 (15.45)	4 (3.33)	NA
Clear cell	20 (18.18)	15 (12.50)	NA
Other	2 (1.82)	5 (4.17)	NA
Non-epithelial	43 (39.09)	20 (16.67)	NA
Sex cord-stromal	35 (31.82)	10 (8.33)	NA
Germ cell	8 (7.27)	10 (8.33)	NA
Lesion characteristics			
Cystic	49 (21.88)	84 (36.52)	4 (26.67)
Solid	107 (47.77)	36 (15.65)	6 (40.00)
Mixed cystic-solid	68 (30.36)	110 (47.83)	5 (33.33)

Data are presented as *n* (%), mean ± standard deviation, or range unless otherwise indicated

NA, no information in the original dataset; TCGA-OV, The Cancer Genome Atlas Ovarian Cancer

**Fig. 3:**
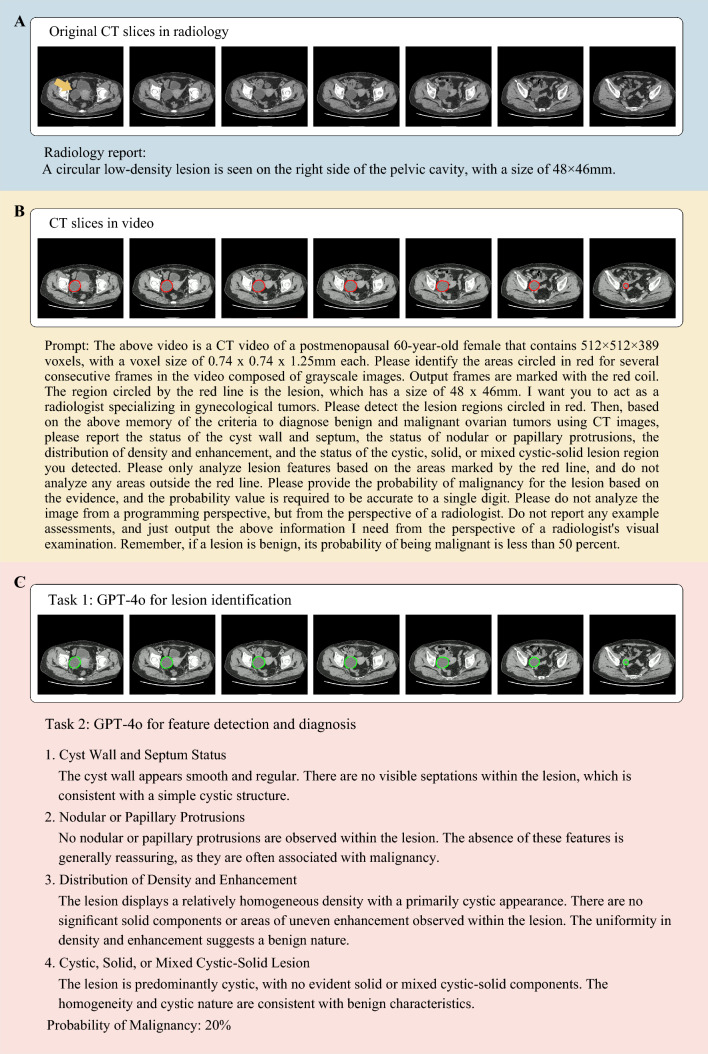
Example of GPT-4o identifying and analyzing computed tomography (CT) images of ovarian mass. **A** Pelvic CT imaging of one participant. **B** The detailed prompt used for GPT-4o diagnosis. **C** GPT-4o identification of the lesion area and the output analysis report about the features of the lesion and the diagnosis of the lesion being benign or malignant

Plain pre-treatment pelvic CT images of 74 patients (15.45%) and contrast-enhanced pelvic CT images of 405 patients (84.55%) were analyzed. A total of 479 CT videos (average duration: 31.00 seconds) were generated. Figure 3 presents the GPT-4o analysis process.

### GPT-4o Evaluation of Ovarian Lesions

Supplementary Fig. S1 illustrates the procedure of GPT-4o identifying lesion regions within pelvic CT videos. Based on the lesion frames output by GPT-4o, GPT-4o successfully identified all the expert-annotated lesion regions (100% [479/479]).

The accuracy for ovarian cancer diagnosis among the experts was as follows: 87.83% (R1), 85.22% (R2), 84.35% (R3), 84.38% (G1), 77.23% (G2), and 75.89% (G3), respectively. Meanwhile, GPT-4o achieved an overall accuracy of 79.96% (80.80%, 79.14%, and 93.33% for the first, second, and TCGA-OV datasets, respectively). For AUC, significant differences were observed between GPT-4o and R1, R2, and R3 (0.80 vs. 0.88, 0.85, and 0.84, DeLong test *p* < 0.05) but not between GPT-4o and G1, G2, and G3 (0.80 vs. 0.85, 0.77, and 0.76, DeLong test *p* > 0.05, as shown in Table 2). Examples GPT-4o identification and diagnosis of benign and malignant are shown in Fig. S2.

**Table 2:** Diagnostic performance of GPT-4o and the six clinicians

Assessor	Accuracy (%)	Sensitivity (%)	Specificity (%)	AUC	Delong test (*p*)
GPT-4o	79.96 (76.03–83.38)	76.83 (71.33–81.56)	84.10 (78.32–88.57)	0.80 (0.76–0.84)	*Ref*
R1	87.83 (82.97–91.44)	85.83 (78.71–90.84)	90.29 (83.04–94.64)	0.88 (0.83–0.92)	0.001*
R2	85.22 (80.05–89.22)	85.71 (78.31–90.89)	84.68 (76.84–90.21)	0.85 (0.81–0.90)	0.02*
R3	84.35 (79.09–88.47)	83.73 (76.22–89.22)	85.05 (77.08–90.58)	0.84 (0.80–0.89)	0.04*
G1	84.38 (79.05–88.55)	80.49 (72.61–86.52)	89.11 (81.54–93.81)	0.85 (0.80–0.89)	0.23
G2	77.23 (71.31–82.24)	78.10 (69.27–84.94)	76.47 (68.10–83.19)	0.77 (0.72–0.83)	0.12
G3	75.89 (69.89–81.03)	74.14 (65.49–81.24)	77.78 (69.06–84.59)	0.76 (0.70–0.82)	0.10

R1–R3 represent radiologists, and G1–G3 represent gynecological oncologists. The GPT-4o evaluation metrics are based on the two hospital datasets; the GPT-4o evaluation results for TCIA are not included in this table because the TCIA dataset only has positive cases

^*^Significant statistical difference; Data in parentheses represent 95% confidence intervals

AUC, area under the receiver operating characteristic curve; TCIA, The Cancer Imaging Archive

### Clinician Evaluation of GPT-4o's Report

Agreement with the experts for GPT-4o feature description was the highest for the distribution of density and enhancement feature (median score 4.67 [IQR 4.00–4.67], mean score 4.30 ± 0.77) across all clinicians. This was followed by the feature cystic, solid, or mixed cystic-solid lesion (median score 4.44 [IQR 4.00–4.67], mean score 4.25 ± 0.64). The model also performed well on nodular or papillary protrusions (median score 4.67 [IQR 3.67–4.67], mean score 4.24 ± 0.81). However, GPT-4o description of the feature cyst wall and septum status was inferior to that of other features (median score 4.67 [IQR 3.67–4.67], mean score 4.22 ± 0.75). Across these four radiological features, GPT-4o achieved an overall average clinician-agreement score of 4.25/5.00. The expert scores for each feature are presented in Table S1 and Fig. S3.

In addition, the inter-radiologist agreement regarding GPT-4o output on the four ovarian cancer-related CT features demonstrated the highest level of consensus on the cystic-solid composition feature (weighted kappa coefficients: 0.45–0.51). However, gynecologic oncologists showed lower agreement on this feature (0.08–0.25). Although radiologists consistently showed higher agreement than oncologists for the remaining three features, all weighted kappa values remained < 0.46, indicating poor agreement (Table S2).

With respect to the clinical utility of the GPT-4o output, the six experts assessed the extent of inappropriate content with an average score of 2.38 ± 0.73. For the extent of missing content, the average score among the six experts was 2.75 ± 0.56. Finally, for the likelihood of possible harm, the average score among the six experts was 2.59 ± 0.72 (for details see Table S3).

### Test–Retest Experiment

In the first test–retest experiment, GPT-4o achieved 90.00% reproducibility, with 45 patients receiving consistent diagnoses (18 benign and 27 malignant) across the two rounds of GPT-4o evaluation. For feature description, the highest reproducibility was for the feature distribution of density and enhancement (79.98% on average), followed by nodular or papillary protrusions (79.64%), and cystic-solid component (79.08%). The lowest reproducibility was for cyst wall and septum status feature (71.62%).

In the second test–retest experiment, the four additional radiologists and gynecologic oncologists achieved diagnostic accuracies of 85.65% (R4), 83.91% (R5), 73.66% (G4), and 67.86% (G5) without GPT-4o assistance in the first round. In the second round, the same four experts achieved diagnostic accuracies of 87.83% (R4), 85.65% (R5), 78.13% (G4), and 84.38% (G5) with GPT-4o assistance. There were significant differences in the AUC between the two rounds for G4 (0.74 vs. 0.78, *p* = 0.003) and G5 (0.68 vs. 0.85, *p* < 0.001) but not for R4 (0.86 vs. 0.88, *p* = 0.23) and R5 (0.84 vs. 0.85, *p* = 0.52).

## Discussion

This study shows that GPT-4o can automatically detect high-risk radiological features associated with early-stage ovarian cancer on pelvic CT images. GPT-4o achieves an average accuracy of 4.25/5.00 in identifying key ovarian cancer-related CT features based on the evaluation of the radiologists and gynecologic oncologists. Additionally, GPT-4o achieves an overall diagnostic accuracy of 79.96% for early-stage ovarian cancer, matching the performance of gynecologic oncologists with 7 years of experience. The experts favorably rated the correctness, completeness, and safety of the GPT-4o output, demonstrating the credibility of its diagnostic assistance. Moreover, GPT-4o significantly improved the diagnostic accuracy ( AUC 0.78–0.85, *p* < 0.01) of the gynecologic oncologists with < 7 years of experience, highlighting its potential for enhancing diagnosis in gynecologic oncology. These findings suggest that GPT-4o can serve as an effective assistive tool for well-trained gynecologic surgeons, particularly in settings where specialized radiologists are not available, enhancing diagnostic accuracy and supporting clinical decision-making in early-stage ovarian cancer.

AI has been recently reported to be potentially useful in ovarian cancer diagnosis by leveraging data from physical examinations, ultrasound images, and CT images.^29–31^ However, most existing AI models provide only a binary classification probability (cancerous or noncancerous) without transparency regarding the underlying computational processes.^32–35^ In addition, traditional machine learning methods and deep learning models such as convolutional neural networks heavily depend on large-scale, accurately annotated datasets. When training samples are limited, these models are prone to overfitting and struggle to capture the complex and subtle features inherent in medical images. The large language model-based approach used in this study overcomes these limitations. Guided by the prompts from the current clinical guidelines and related studies,^8–11^ GPT-4o first identified high-risk imaging features associated with ovarian cancer within the primary lesion and then leveraged this information to generate a diagnosis. This two-step process enhanced the transparency and interpretability of GPT-4o’s decision making. Further, clinicians can directly trace how the model identifies specific imaging features and rationalizes its way to a diagnostic conclusion, increasing confidence in its output. Moreover, the GPT-4o-assisted diagnostic approach potentially democratizes access to AI-powered tools for ovarian cancer diagnosis. This eliminates the need for specialized engineering knowledge in the design of complex neural network architectures. Instead, clinicians can be assisted by readily available screen-recording tools to capture CT scan videos as input for GPT-4o analysis. This accessibility has the potential to break down interdisciplinary barriers and pave the way for a more convenient and efficient digital medicine workflow for ovarian cancer.

Accurate image interpretation is important for early detection of ovarian cancer. In this study, GPT-4o demonstrated high concordance with experts in identifying the feature cystic-solid components. We hypothesize that this may be because GPT-4o primarily relies on gray-scale image differences to recognize cystic and solid areas, and the CT values of cystic versus solid masses differ substantially, making this feature relatively easier for the model to identify. Conversely, the model exhibited the lowest concordance with expert assessment in recognizing the feature cyst wall and septum status. This discrepancy may reflect the model’s challenges in detecting subtle differences in these structures, potentially due to limitations in its training on medical imaging data and the inherent difficulties of CT imaging, including noise, limited contrast, and artifacts. These explanations are hypotheses based on our understanding of the visual characteristics of these features and should not be interpreted as definitive insights into the internal workings of GPT-4o. Future applications of GPT-4o in clinical ovarian cancer diagnosis should prioritize pre-training on cyst wall and septum status recognition and enhancing the reliability of reporting subtle ovarian-specific features. To mitigate these challenges, it is essential to increase the training sample size by acquiring more diverse data from multiple hospitals or imaging centers, ensuring robust generalizability across different devices and imaging conditions. Additionally, incorporating advanced preprocessing techniques—such as contrast enhancement, noise suppression, and edge sharpening—can help preserve fine details.

This study had some limitations. First, although manually outlining the boundaries of the primary ovarian mass in the CT videos ensured accurate lesion identification with GPT-4o, this approach introduced a level of manual intervention comparable to that used in previous radiomics and deep learning studies.^36–38^ The contours were generated in ITK-SNAP, and future work should evaluate fully automated, end-to-end lesion-detection pipelines to minimize operator bias. Second, external validation relied on the TCGA-OV cohort, the only publicly accessible dataset currently available to us that includes CT scans from patients with early-stage ovarian cancer. Notably, the TCGA-OV cohort does not include benign ovarian lesions. The limited sample size in the current dataset may not adequately represent the full spectrum of pathological features and patient conditions, leading to the underrepresentation of certain subpopulations. This limitation restricts the model's applicability in diverse clinical settings, underscoring the necessity of incorporating multicenter data to validate and enhance the performance of GPT-4o. Third, the pathological types of ovarian masses are complex, and the key imaging features of benign and malignant ovarian masses are not generalizable for all these pathological types. Future studies should incorporate more detailed CT imaging features corresponding to each ovarian mass pathology type for GPT-4o training. Finally, this study exclusively employed CT images as inputs. Further studies should explore multimodal diagnostic strategies that integrate ultrasound and MRI images with physical examination indicators.

In addition to these methodological and data-related limitations, the use of general-purpose large language models introduces additional considerations regarding the reliability and clinical trustworthiness of model outputs. In this study, six experts evaluated the model’s outputs across three domains: the extent of inappropriate content, the extent of missing content, and the likelihood of possible harm. Overall, the risk in these domains was relatively low; nevertheless, the potential for incomplete or clinically inappropriate outputs remains a central challenge when deploying general-purpose large language models in medical practice. Such outputs may arise from inherent knowledge constraints of general-purpose large language models, potential hallucination effects, limitations in the training data, or insufficiently specific prompts, which may lead to omissions of critical information or mischaracterization of imaging features. These inaccuracies could mislead clinicians if interpreted without expert oversight, potentially affecting diagnosis or treatment planning. To mitigate these risks, structured prompts, explicit verification of key clinical features, and review by experienced radiologists or gynecologic oncologists are essential. Incorporating these safeguards ensures that the vision language models function as an assistive tool rather than a replacement for clinical judgment. Moving forward, systematic evaluation of output reliability and continuous monitoring of potential risks will be crucial for the safe and trustworthy integration of large language models into diagnostic workflows.

In conclusion, GPT-4o can automatically identify key CT features associated with ovarian cancer. GPT-4o achieves promising diagnostic accuracy comparable to that of experts in detecting these features, demonstrating its potential to enhance the accuracy of gynecologic oncologist diagnoses. With further validation in diverse datasets, GPT-4o holds promise as a novel approach for early ovarian cancer detection, ultimately improving the current landscape of early-stage ovarian cancer management.

## Supplementary Information

Below is the link to the electronic supplementary material.

Supplementary file1 (DOCX 15618 KB)

## Data Availability

The CT videos, and results of the open-access dataset have been uploaded to Mendeley Data: 10.17632/r3bxp8mskj.1.
