# Supplementary material for: A Novel Approach to Ovarian Cancer Diagnosis via CT Imaging: GPT-4o-Driven Automated Feature Recognition and Validation in Clinical Settings
Source: Ann Surg Oncol. 2026 Feb 17;33(7):6618–28. doi: 10.1245/s10434-026-19248-2 (PMC13242454; doi:10.1245/s10434-026-19248-2)
Supplement: Supplementary file 1 — Supplementary file1 (DOCX 15618 KB) [file 10434_2026_19248_MOESM1_ESM.docx]

**Supplementary A1**

I want you to act as a radiologist specializing in gynecological tumors. Please remember the following criteria for diagnosing benign and malignant ovarian cancer using CT images. Benign ovarian tumors typically present the following characteristics: (1) cystic lesions with a smooth cyst wall. (2) Any septum observed is uniformly thick, and if there's a solid component, it usually displays a uniform density, often exhibiting a homogeneous pattern of enhancement. In contrast, malignant ovarian tumors often exhibit the following characteristics: (1) predominantly cystic-solid or solid structures. (2) The septum or cystic wall may show nodular or papillary protrusions and uneven thickness, especially thickening exceeding 3mm in the most pronounced areas. (3) Solid components typically display uneven density with a notable presence of blood supply, leading to uneven enhancement patterns.

**Supplementary A2**

The above video is a CT video of a premenopausal 35-year-old female that contains 512*512*96 voxels, with a voxel size of 0.79*0.79*5.00mm each. Please identify the areas circled in red for several consecutive frames in the video composed of grayscale images. Output frames are marked with the red coil. The region circled by the red line is the lesion, which has a size of 97mm×88mm. I want you to act as a radiologist specializing in gynecological tumors. Please detect the lesion regions circled in red. Then, based on the above memory of the criteria to diagnose benign and malignant ovarian tumors using CT images, please report the status of the cyst wall and septum, the status of nodular or papillary protrusions, the distribution of density and enhancement, and the status of the cystic, solid, or mixed cystic-solid lesion region you detected. Please only analyze lesion features based on the areas marked by the red line, and do not analyze any areas outside the red line. Please provide the probability of malignancy for the lesion based on the evidence, and the probability value is required to be accurate to a single digit. Please do not analyze the image from a programming perspective, but from the perspective of a radiologist. Do not report any example assessments, and just output the above information I need from the perspective of a radiologist's visual examination. Remember, if a lesion is benign, its probability of being malignant is less than 50 percent.


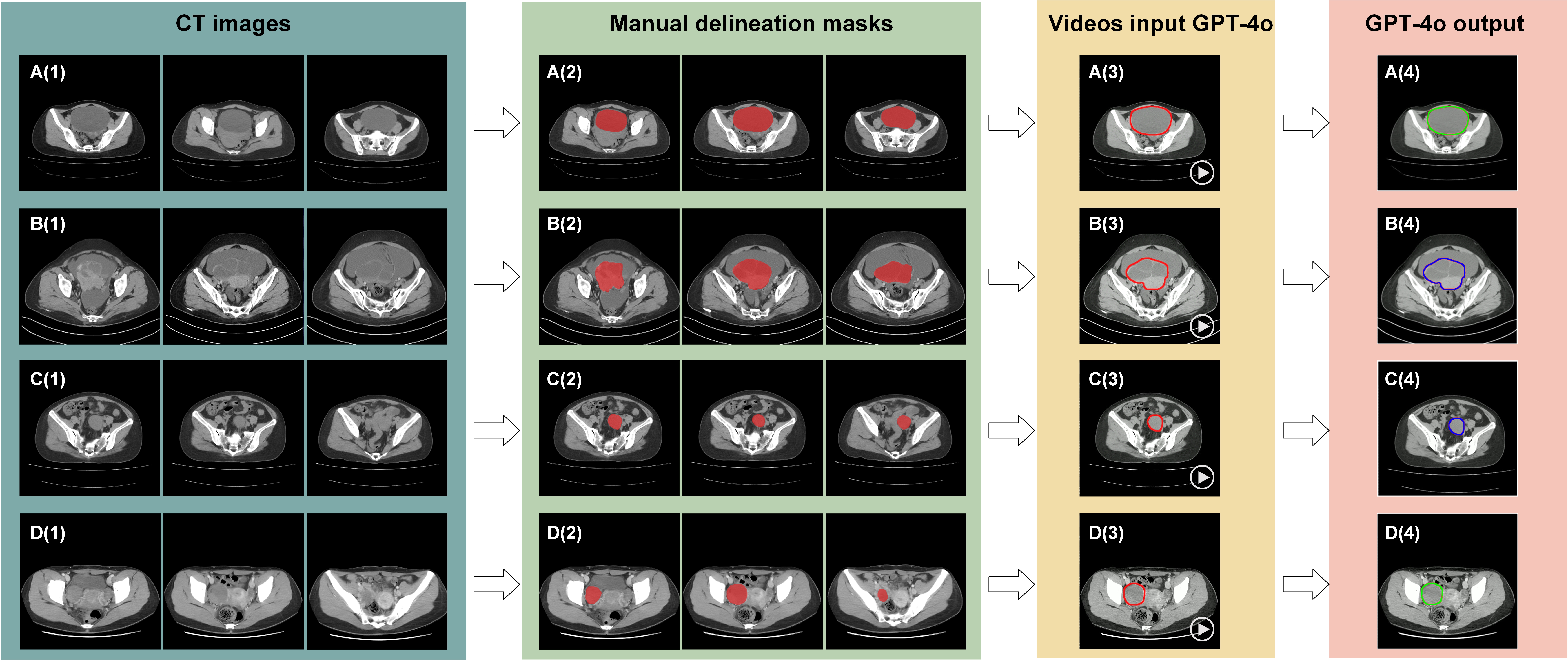


**Figure S1.** Illustration of the procedure of GPT-4o identifying the manual delineation masks of the ovarian masses. (1) Pelvis CT images obtained from the picture acquisition and communication system. (2) Manual delineation masks generated by the experts manually segmenting the pelvic adnexal masses using ITK-SNAP software. (3) Conversion of the manually segmented images to CT video using a video converter. The boundaries of the masses were outlined by red with a width of 5 pixels on the slices. (4) GPT-4o generated delineations of the pelvic adnexal masses it identified within the input video sequences. The color selection for these delineations was determined autonomously by GPT-4o, without requiring explicit instructions within the prompt.


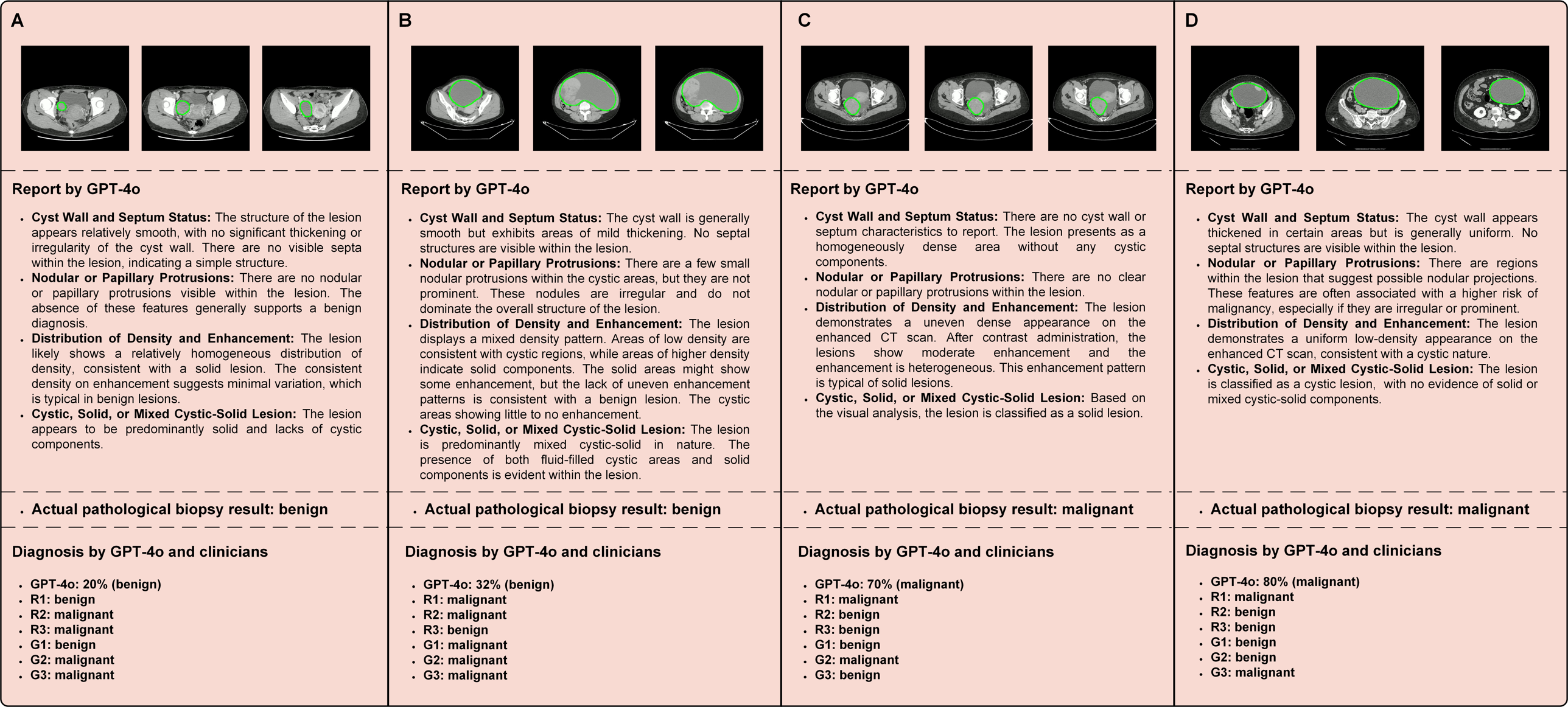


**Figure S2** Examples GPT-4o identification and diagnosis of benign and malignant.


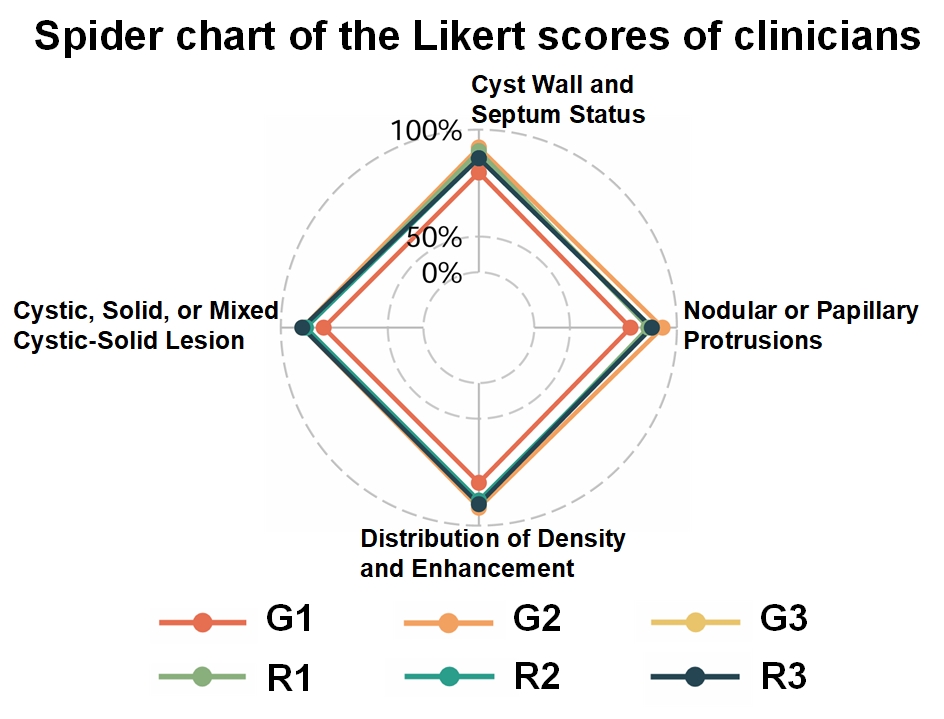


**Figure S3.** Spider chart of the average Likert scores from the clinician's rating of the image features of the GPT-4o output. R1-R3 represent radiologists, G1-G3 represent gynecological oncologists.

**Table S1.** Clinician scores for each feature

|  |  | **Cyst Wall and Septum Status** | **Nodular or Papillary Protrusions** | **Distribution of Density and Enhancement** | **Cystic, Solid, or Mixed Cystic-Solid Lesion** |
| --- | --- | --- | --- | --- | --- |
| **R1** | Median (IQR) | 5.00 (4.00-5.00) | 5.00 (3.00-5.00) | 5.00 (4.00-5.00) | 5.00 (4.00-5.00) |
| **R2** | Median (IQR) | 4.00 (4.00-5.00) | 5.00 (4.00-5.00) | 5.00 (4.00-5.00) | 5.00 (4.00-5.00) |
| **R3** | Median (IQR) | 5.00 (3.00-5.00) | 5.00 (4.00-5.00) | 5.00 (4.00-5.00) | 5.00 (4.00-5.00) |
| **G1** | Median (IQR) | 4.00 (4.00-4.00) | 4.00 (4.00-4.00) | 4.00 (4.00-4.00) | 4.00 (4.00-4.00) |
| **G2** | Median (IQR) | 5.00 (4.00-5.00) | 5.00 (5.00-5.00) | 5.00 (5.00-5.00) | 5.00 (4.00-5.00) |
| **G3** | Median (IQR) | 5.00 (3.00-5.00) | 5.00 (4.00-5.00) | 5.00 (4.00-5.00) | 5.00 (4.00-5.00) |

R1-R3 represent radiologists, G1-G3 represent gynecological oncologists. SD: standard deviation, IQR: interquartile range.

**Table S2**. Agreement (weighted Kappa coefficients) among clinician scores for the four key ovarian cancer-related CT features.

| **Weighted Kappa (κ)** | **Cyst Wall and Septum Status** | **Nodular or Papillary Protrusions** | **Distribution of Density**  **and Enhancement** | **Cystic, Solid, or Mixed Cystic-Solid Lesion** |
| --- | --- | --- | --- | --- |
| **Gynecologic oncologists** |  |  |  |  |
| G1 vs. G2 | 0.08 (0.02-0.14) | 0.09 (0.04-0.14) | 0.09 (0.04-0.15) | 0.08 (0.03-0.14) |
| G2 vs. G3 | 0.18 (0.08-0.30) | 0.18 (0.06-0.29) | 0.26 (0.13-0.38) | 0.25 (0.12-0.38) |
| G1 vs. G3 | 0.15 (0.09-0.21) | 0.17 (0.10-0.24) | 0.12 (0.05-0.18) | 0.14 (0.08-0.21) |
| **Radiologists** |  |  |  |  |
| R1 vs. R2 | 0.35 (0.25-0.44) | 0.39 (0.30-0.48) | 0.36 (0.26-0.46) | 0.45 (0.34-0.53) |
| R2 vs. R3 | 0.40 (0.30-0.49) | 0.44 (0.33-0.55) | 0.46 (0.37-0.55) | 0.51 (0.41-0.60) |
| R1 vs. R3 | 0.40 (0.30-0.50) | 0.32 (0.23-0.42) | 0.38 (0.28-0.48) | 0.46 (0.34-0.56) |

R1-R3 represent the three radiologists, G1-G3 represent the three gynecological oncologists.

**Table S3.** Clinician evaluation of clinical utility of the GPT-4o output. The numbers are mean value with standard deviation.

|  | The extent of inappropriate content | The extent of missing content | The likelihood of possible harm |
| --- | --- | --- | --- |
| **All** | 2.38 (0.73) | 2.75 (0.56) | 2.59 (0.72) |
| **R1-R3** | 2.25 (0.81) | 2.60 (0.70) | 2.42 (0.83) |
| **G1-G3** | 2.51 (0.62) | 2.91 (0.28) | 2.76 (0.55) |

R1-R3 represent radiologists, G1-G3 represent gynecological oncologists.
